# Fingolimod Promotes Antibacterial Effect of Doripenem against Carbapenem-Resistant *Escherichia coli*

**DOI:** 10.3390/antibiotics11081043

**Published:** 2022-08-02

**Authors:** Hye-Won Jin, Hye-Rim Kim, Yong-Bin Eom

**Affiliations:** 1 Department of Biomedical Laboratory Science, College of Medical Sciences, Soonchunhyang University, Asan 31538, Korea; jhy3682@naver.com; 2 Department of Medical Sciences, Graduate School, Soonchunhyang University, Asan 31538, Korea; goa6471@naver.com

**Keywords:** fingolimod, doripenem, carbapenem, carbapenemase, *Escherichia coli*, synergistic action

## Abstract

The aim of this study was to determine whether fingolimod could synergize the antibacterial activity of doripenem against carbapenem-resistant *Escherichia coli* (CREC) and its potential as an antibiotic adjuvant for doripenem. The *E. coli* used in this study had the *bla_KPC_* gene and became resistant to many classes of antibiotics, particularly carbapenems. The minimum inhibitory concentrations (MICs) of fingolimod and doripenem were determined. To investigate the synergistic action between fingolimod and doripenem, synergy checkerboard, growth curve, and time-kill analyses were performed. A motility test was also performed using a semi-solid medium to determine whether fingolimod could inhibit the motility of *E. coli*, one of its virulence mechanisms. The expression levels of carbapenemase-, motility-, and efflux pump-related genes suppressed by fingolimod were analyzed by quantitative polymerase chain reaction (qPCR). Our study demonstrated that the combination of fingolimod and doripenem inhibited carbapenemase, biological activity and other CREC virulence factors. This study findings suggest the potential of fingolimod as an adjuvant to prevent antibiotic resistance in CREC.

## 1. Introduction

*Escherichia coli* is the most commensal inhabitant of the gastrointestinal tract of humans and warm-blooded animals [1]. It is a normal bacterium that has coexisted and mutually benefitted human health for decades [2]. However, sometimes, *E. coli* causes diseases such as cystitis, urinary tract infections, appendicitis, peritonitis, and pneumonia [3,4,5]. Recent data show rapidly increasing rates of infections by multidrug-resistant (MDR) gram-negative bacilli due to their resistance to carbapenems, resulting in high morbidity and mortality [6].

Carbapenems are known as the last resort for treating gram-negative bacteria such as *E. coli*. The emergence of *E. coli* resistant to these antibiotics further increases the risk of multidrug resistance. Carbapenemase-producing *Enterobacteriaceae* (CPE) was almost nonexistent until the 1990s. However, CPE is encountered routinely in hospitals and other healthcare facilities in many countries today [7]. Carbapenemase is a β-lactamase with broad hydrolytic activity in multidrug-resistant gram-negative bacteria [8]. β-lactamases are grouped into Ambler classes A, B, and C [8]. The most dangerous β-lactamase spreading worldwide is *bla_KPC_* [9]. The production of carbapenemases is one of the main mechanisms for the resistance of carbapenem-resistant *Escherichia coli* (CREC) strains. However, some *E. coli* do not produce carbapenemase, possessing alternative CREC mechanisms, which include efflux pump overexpression and mutation or the loss of outer membrane porin [10].

There are five families of MDR efflux pumps: (1) the resistance-nodulation-division (RND) family; (2) the small multidrug resistance (SMR) family; (3) the major facilitator superfamily (MFS); (4) the multidrug and toxic compound extrusion (MATE) family; and (5) the ATP (adenosine triphosphate)-binding cassette (ABC) superfamily [11]. Among these, the pumps in the RND family are the only pumps related to the outer membrane and the inner membrane. They play an important role in inducing the multidrug resistance of gram-negative bacteria. The RND efflux pump is a tripartite complex composed of a membrane fusion protein, a cytoplasmic membrane, and an outer membrane protein. In *E. coli*, the gene associated with membrane fusion protein is *acrA*, the gene associated with the outer membrane protein is *tolC*, and the genes associated with the cytoplasmic membrane are *acrB* and *acrD* [11].

Fingolimod used in this study was the first oral drug approved by the United States Food and Drug Administration (US FDA) for the treatment of multiple sclerosis [12]. Fingolimod is a sphingosine-1-phosphate receptor modulator that can reduce lymphocyte recycling from lymph nodes to the blood and peripheral tissues, including inflammatory lesions and transplant sites [13]. The use of treatments that have already been approved has two advantages: (1) the safety of the drug has been proven and (2) the use of approved drugs is more efficient than developing new drugs because of the time saved. In addition, previous studies have shown that fingolimod has an antibacterial effect [14]. 

Although the antibacterial activity of fingolimod has been demonstrated, studies examining other possible synergistic actions between fingolimod and carbapenem antibiotics against CREC have not yet been reported. Therefore, the purpose of the present study was to evaluate the potential of using fingolimod as an adjuvant drug to treat infections caused by CREC.

## 2. Results

### 2.1. Detection of Carbapenem-Resistant Escherichia coli

The minimum inhibitory concentration (MIC) of fingolimod and carbapenem antibiotics (doripenem, meropenem, imipenem, and ertapenem) against clinically isolated *E. coli* strains (KBN12P05816, KBN12P05795, and KBN12P06081) was determined. The results are shown in Table 1. The resistance criteria of carbapenems were determined according to Clinical and Laboratory Standards Institute (CLSI) breakpoints [15]. Polymerase chain reaction (PCR) analysis confirmed that the clinically isolated *E. coli* strains contained the carbapenemase *bla_KPC_* gene. The findings demonstrate that the clinically isolated *E. coli* strains were resistant to carbapenem, identifying them as CREC.

### 2.2. Synergistic Action of Fingolimod in Combination with Doripenem

**Figure 1:**
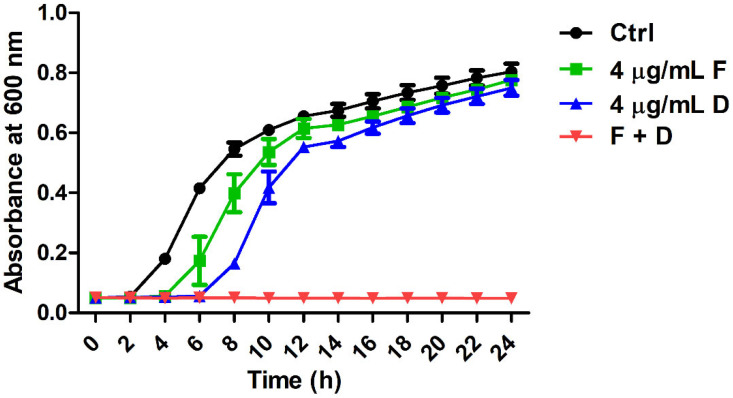
The growth of *E. coli* KBN12P05816 was analyzed in the presence of fingolimod and doripenem. Each sample was plated on trypticase soy broth (TSB) plates and incubated at 37 °C for 24 h. (●) Untreated controls, (■) 4 μg/mL of fingolimod, (▲) 4 μg/mL of doripenem, and (▼) the combination of 4 μg/mL of fingolimod and 4 μg/mL of doripenem.

**Figure 2:**
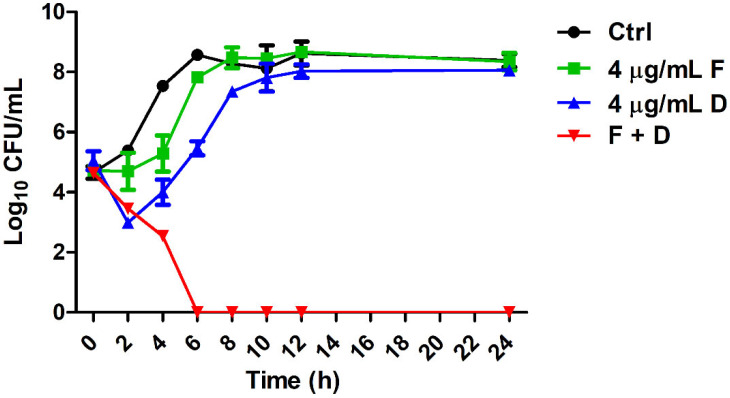
Synergistic action of fingolimod and doripenem against CREC. *E. coli* KBN12P05816 was treated with fingolimod and doripenem, plated with a spreader on Muller–Hinton agar (MHA), and incubated at 37 °C for 24 h. The CFU/mL values were recorded. (●) Untreated controls, (■) 4 μg/mL of fingolimod, (▲) 4 μg/mL of doripenem, and (▼) the combination of 4 μg/mL of fingolimod and 4 μg/mL of doripenem.

To investigate the synergistic action of fingolimod and doripenem on CREC, growth curve, checkerboard, and time-kill analyses were performed. The time-dependent synergistic action of *E. coli* KBN12P05816 with high resistance to carbapenem was analyzed (Table 2, Figure 1 and Figure 2). As shown in Table 2, the combination of fingolimod and doripenem decreased the MIC for CREC compared to either fingolimod or doripenem alone. The fractional inhibitory concentration index (FICI) value was 0.3125. The FICI value of 0.5 or less indicates synergistic action. Therefore, the combination of fingolimod and doripenem acted synergistically on *E. coli* highly resistant to carbapenem. Comparing the control group containing DMSO instead of fingolimod and doripenem at a concentration of 4 μg/mL, doripenem hardly inhibited the growth of *E. coli*. Similarly, fingolimod at 4 μg/mL resulted in a slight delay in growth. In contrast, the combination of fingolimod and doripenem notably inhibited the growth of *E. coli* (Figure 1 and Figure 2).

### 2.3. Inhibitory Effect of Fingolimod on Motility

**Figure 3:**
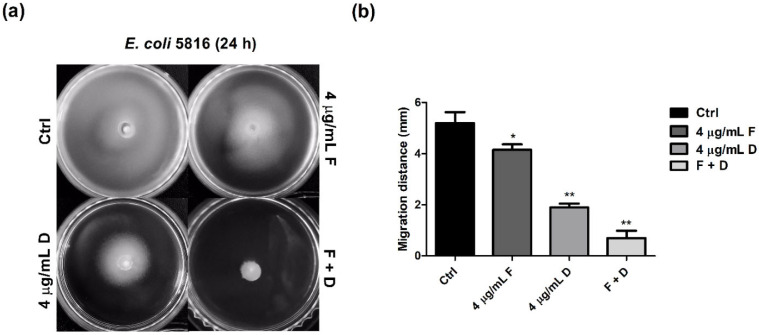
Effect of fingolimod and doripenem on the motility of CREC. (**a**) The motility of CREC KBN12P05816 was analyzed on semisolid agar plates containing fingolimod and doripenem. (**b**) Bar graph showing the mean migration distance (mm). The decrease in migration distance was significant (* *p* < 0.05 and ** *p* < 0.01).

A phenotypic study was performed to investigate the effect of fingolimod on CREC motility. In Figure 3, the migration distance was significantly inhibited after 24 h of incubation. Compared to the controls, the combination of fingolimod and doripenem inhibited the motility of CREC.

### 2.4. Fingolimod Suppressed the Expression of Genes Associated with Antibiotic Resistance in E. coli

**Figure 4:**
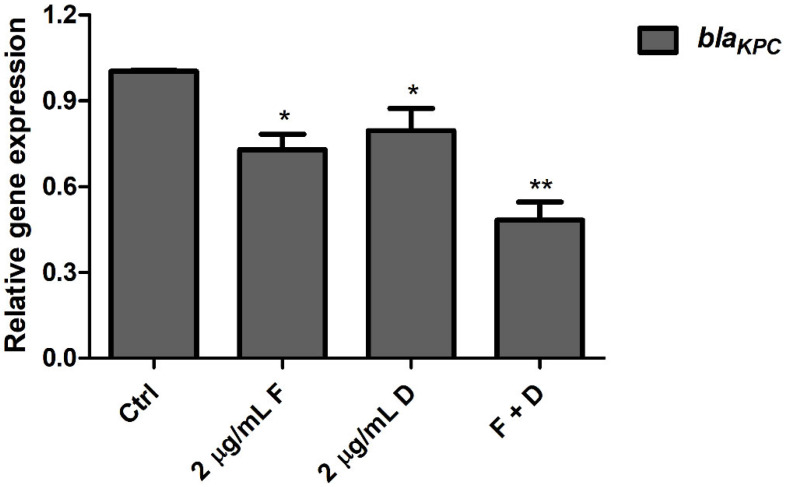
Transcriptional changes involving the *bla_KPC_* gene in CREC. After treatment with fingolimod alone (2 μg/mL F), doripenem alone (2 μg/mL D), or the combination of 2 μg/mL of doripenem and 2 μg/mL of fingolimod (F + D), qPCR analysis of the *bla_KPC_* gene in *E. coli* KBN12P05816 was performed. *16S rRNA* was used to normalize the transcriptional levels of the target gene. The error bars represent the means and standard deviation (SDs). Asterisks (*) indicate statistically significant differences (* *p* < 0.05 and ** *p* < 0.01) from the controls.

**Figure 5:**
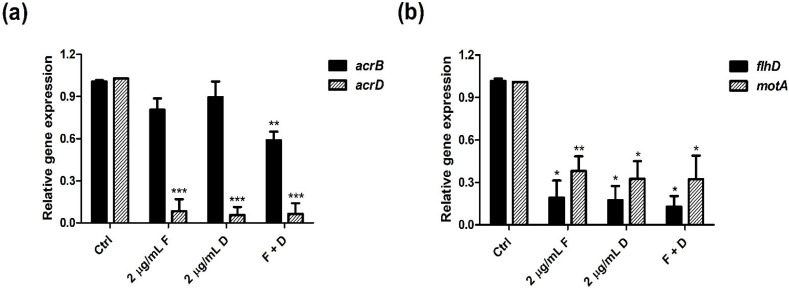
Transcriptional changes in virulence-related genes in *E. coli*. Expression levels of (**a**) efflux pump-related (*acrB*, *acrD*) genes and (**b**) motility-related (*flhD*, *motA*) genes are presented with a histogram. After treatment with fingolimod alone (2 μg/mL F), doripenem alone (2 μg/mL D), or the combination of 2 μg/mL of doripenem and 2 μg/mL of fingolimod (F + D), transcription levels of the target genes were normalized to that of *16S rRNA*. Asterisks (*) indicate a significant decrease in expression levels in each gene (* *p* < 0.05 and ** *p* < 0.01, and *** *p* < 0.001).

The qPCR results indicate that the expression levels of carbapenemase-, motility-, and efflux pump-related genes were reduced by the combination of fingolimod with doripenem compared to treatment with either fingolimod or doripenem alone. As shown in Figure 4, fingolimod inhibited the carbapenemase-related *bla_KPC_* gene more effectively than doripenem. The combination of doripenem and fingolimod resulted in a significant decrease in its expression compared to the controls. These results demonstrate that fingolimod inhibited carbapenemase production in CREC and its actions were synergistic with doripenem. More specifically, among the efflux pump-related genes *acrB* and *acrD*, the expression level of the *acrD* gene was significantly downregulated compared to the controls. *acrB* gene expression was slightly reduced by fingolimod and doripenem at sub-MICs (Figure 5a). The expression levels of motility-related genes (*flhD, motA*) were decreased by treatment with sub-MICs of fingolimod and doripenem alone or in combination (Figure 5b). Taken together, these results suggest that the combination of fingolimod and doripenem could decrease the expression of carbapenemase-, efflux pump-, and motility-related genes. In addition, the suitability of the reference gene *16S rRNA* used as normalization in this study was validated, referring to the MIQE guidelines [16].

## 3. Discussions

With the continued use of antibiotics, the rate of MDR is increasing. In particular, resistance to carbapenem antibiotics used as the last line of defense against *Enterobacteriaceae* infections is a worldwide problem [17]. Recently, there has been growing interest in finding synergistic adjuvants that can solve the problem of MDR [6]. To deter the emergence and spread of antibiotic-resistant bacteria, antibacterial research to identify new antibiotic adjuvants is considered urgent.

The resistant bacteria used in our study had acquired the *bla_KPC_* gene encoding the *Klebsiella pneumoniae* carbapenemase (KPC) enzyme. They belong to Ambler class A. A previous study demonstrated that a high percentage of *E. coli* and *K. pneumoniae* had the *bla_KPC_* gene [18]. Similar to that study, the present study also confirmed by polymerase chain reaction that the clinical isolate (*E. coli* KBN12P05816) used in this study expressed the *bla_KPC_* gene.

A previous study demonstrated that the antifungal compound myriocin had potent activity against yeast and dermatophytes in vitro [19]. Fingolimod is derived from myriocin, a metabolite of *Myriococcum albomyces*. It is used to treat recurrent forms of multiple sclerosis (MS). However, few studies have reported the role of fingolimod as an antibiotic adjuvant despite its potent activity against yeast and dermatophytes. Therefore, in this study, we used the checkerboard method and demonstrated that there was synergistic action between fingolimod and carbapenem antibiotics at FICIs < 0.35 (Table 2). The combination of fingolimod and doripenem made CREC strains sensitive to doripenem.

The rate and extent of bacterial growth and synergistic action were evaluated over time by growth curves (Figure 1) and time-killing assays (Figure 2). The growth curves showed that the bacteria did not grow from the beginning of the culture. In the time-kill assay, the death of the bacteria was confirmed from 6 h after incubation. This indicates that fingolimod not only has the ability to prevent the initial growth of the bacteria but also could affect already formed colonies.

Motility is one of the most important features of *Enterobacteriaceae* and many other bacteria. In this study, we demonstrated that fingolimod could inhibit the motility of CREC at the phenotypic level (Figure 3). Motility-related genes (*flhD*, *motA*) were also significantly reduced by fingolimod, validating the genotypic effect of fingolimod (Figure 5b). At 4 μg/mL of fingolimod, the migration distance of the bacteria was slightly reduced compared to the controls. This suggests that fingolimod itself could also inhibit CREC motility. In addition, the combination of fingolimod and doripenem significantly reduced the movement distance compared to the controls. These data show that the synergistic action of fingolimod and doripenem also affected motility.

A previous study revealed that of the five RND transporters in *E. coli*, only *acrB*, *acrD*, and *mdtABC* played a role in enterobactin excretion [20]. *acrD*, with 66% homology to *acrB*, has a limited range of amphiphiles, as well as resistance to aminoglycosides [21,22]. That is, when *acrB* and *acrD* are expressed in *E. coli*, *acrD*-expressing bacteria are more resistant to specific anionic beta-lactam antibiotics than *acrB*-expressing bacteria. Thus, differences in beta-lactam resistance are most likely determined by *acrB* and *acrD* [23]. Therefore, in this study, we measured the gene expression levels of *acrB* and *acrD*, which are efflux pump-related genes with similar structures and functions (Figure 5a).

As shown in Figure 5a, the expression level of the *acrB* gene was slightly decreased when fingolimod and doripenem were administered alone. However, it was significantly decreased in the group treated with the combination of fingolimod and doripenem compared to the control group. This suggests that the combination of fingolimod and doripenem had a synergistic action on the expression of the *acrB* gene. However, in the case of *acrD*, its expression was reduced similarly in the group treated with fingolimod and doripenem alone and the antibiotics in combination. For the *acrD* gene, synergy was not seen with the combination of fingolimod and doripenem, suggesting that fingolimod was more effective against *acrD*, which is more resistant to beta-lactam. At the same time, it suggests that fingolimod could exert antibacterial action even when used alone.

In conclusion, this study proved the synergistic action of fingolimod and doripenem on CREC. In addition, we demonstrated a decrease in the expression level of the carbapenemase gene of *E. coli*. Motility, one important virulence factor, was also reduced by fingolimod. Therefore, fingolimod showed the potential as an anti-bacterial and anti-virulence adjuvant. A limitation of our study was that the impact of fingolimod was demonstrated in vitro only. Therefore, an in vivo investigation is necessary to verify the interaction between fingolimod and doripenem.

## 4. Materials and Methods

### 4.1. Organisms, Growth Conditions, and Reagents

Clinical isolates (KBN12P05816. KBN12P05795, and KBN12P06081) were supplied by the Gyeongsang National University Hospital Branch of the National Culture Collection for Pathogens (GNUH-NCCP, Jinju, Korea). These strains were incubated at 37 °C in trypticase soy broth (TSB; Difco, Becton, Dickinson, and Company, Sparks, MD, USA) and sub-cultured on MacConkey agar (MAC; Difco, Becton, Dickinson and Company, Sparks, MD, USA). Doripenem was purchased from Cayman Chemical (Ann Arbor, MI, USA) and dissolved in dimethyl sulfoxide (DMSO). In all analyses, the final concentration of DMSO used as the control was less than 2%.

### 4.2. Minimum Inhibitory Concentration Assay

Minimum inhibitory concentrations (MICs) were measured to determine the antimicrobial susceptibility of *E. coli* to fingolimod and carbapenems. For the MICs, we used the broth microdilution method, referring to the standard procedure of the Clinical and Laboratory Standards Institute (CLSI) [15]. For the MIC assay, 96-well microtiter plates (BD Falcon^TM^, BD, NJ, USA) were used. Absorbance was measured at a wavelength of 600 nm (A_600_) using a Multiskan GO plate reader (Thermo Fisher Scientific, Waltham, MA, USA). The MIC value was defined as the lowest concentration of the antimicrobial agent that inhibited visible cell growth.

### 4.3. DNA Extraction and Polymerase Chain Reaction (PCR)

Genomic DNA was extracted from *E. coli* using a QIAamp DNA Minikit (Qiagen, Valencia, Santa Clarita, CA, USA) according to the manufacturer’s instructions. The concentration and purity of the DNA samples were analyzed using a BioDrop *μ*LITE (BioDrop Ltd., Cambridge, UK). The PCR mixture included 2 μL of 10× PCR buffer (Perkin Elmer, Waltham, MA, USA), 1.2 μL of MgCl_2_ (25 mM; Perkin Elmer), 1.6 μL of dNTPs (2.5 mM; TakaraBio, Shiga, Japan), 2 μL of each primer (10 pmoles/μL; BioNics, Seoul, Korea), 0.25 μL of Ampli Taq Gold (5 U/μL; Perkin Elmer, Waltham, MA, USA), 2 μL of extracted DNA. The final volume was filled to 20 μL with sterile distilled water (D.W). The amplification conditions were as follows: initial denaturation step at 95 °C for 10 min, 30 cycles of denaturation at 95 °C for 1 min, primer annealing at each annealing temperature for 1 min, and extension at 72 °C for 1 min, followed by a final elongation step at 72 °C for 3 min. The primers used in this study are listed in Table 3. Amplified PCR products were separated on 1.5% agarose gel by electrophoresis and then visualized with a Gel Doc system (Bio-Rad, Hercules, CA, USA).

### 4.4. Synergy Checkerboard Assay

A synergy checkerboard experiment was performed in a 96-well microtiter plate to evaluate the synergistic action between fingolimod and doripenem, as described previously [30]. Briefly, the bacterial culture was suspended in trypticase soy broth (TSB) at a final concentration of 1 × 10^6^ CFU/mL and then distributed into 96-well plates at 200 μL per well. The combination of fingolimod and doripenem used a two-fold serial dilution method. The absorbance of the plate incubated at 37 °C for 24 h was measured at a wavelength of 600 nm. The fractional inhibitory concentration index (FICI) was calculated by the formula below [31]. The FIC index (FICI) is the sum of each FIC. The FICI value of ≤0.5 indicates synergistic action, 0.5–4.0 indicates no interaction, and the FICI of ≥4.0 indicates antagonism.

(a)
$FIC\;of\;fingolimod=\frac{MIC\;of\;fingolimod\;in\;combination}{MIC\;of\;fingolimod\;alone}$

(b)
$FIC\;of\;doripenem=\frac{MIC\;of\;doripenem\;in\;combination}{MIC\;of\;doripenem\;alone}$

### 4.5. Time-Kill Assay

To evaluate the synergistic action of doripenem and fingolimod on *E. coli* resistant to carbapenem antibiotics, a time-kill assay was performed. Briefly, bacterial suspensions (1 × 10^6^ CFU/mL) in TSB were exposed to 4 μg/mL of fingolimod, 4 μg/mL of doripenem, and the combination of 4 μg/mL of doripenem and 4 μg/mL of fingolimod and incubated at 37 °C for 24 h. Every 2 h (up to 24 h), each sample was diluted with saline and inoculated onto Muller–Hinton agar (MHA; Difco, Becton, Dickinson and Company, Sparks, MD, USA) plates using a spreader. After 24 h of incubation at 37 °C, the colony-forming units (CFUs) of each sample were recorded.

### 4.6. Motility Inhibition Assay

To evaluate motility, a virulence factor, an agar plate composed of Luria Bertani (LB; Difco, Becton, Dickinson and Company, Sparks, MD, USA) medium and 0.2% agar was used. After adding 4 μg/mL of fingolimod, 4 μg/mL of doripenem, or 4 μg/mL fingolimod +4 μg/mL doripenem to the LB medium and culturing *E. coli* at 37 °C for 24 h, 5 μL of the grown culture (1 × 10^6^ CFU/mL) was inoculated into the center of a motility agar plate. After 24 h of incubation at 37 °C, the migration distance was measured.

### 4.7. RNA Isolation and Quantitative PCR (qPCR)

CREC was grown under the influence of fingolimod and doripenem in TSB until it reached the exponential phase. Cells were collected by centrifuging 2 mL of bacterial suspensions from each sample at 25,000 *g* for 10 min at 4 °C. For RNA isolation and purification, a NucleoSpin RNA Mini Kit (Macherey-Nagel, Düren, Germany) was used, following the manufacturer’s instructions. The BioDrop μLITE (BioDrop Ltd., Cambridge, UK) was used to measure the quality and concentration of the isolated RNA samples. cDNA was synthesized using ReverTraAce qPCR RT Master Mix with gDNA Remover (TOYOBO, Osaka, Japan) according to the manufacturer’s protocols. TOPreal^TM^ qPCR 2X PreMIX (Enzynomics, Daejeon, Korea) was used to amplify cDNA in a StepOnePlus Real-Time PCR System (Applied Biosystems, Foster City, CA, USA). The qPCR cycling conditions were as follows: initial denaturation step at 95 °C for 10 min, followed by 40 cycles of denaturation at 95 °C for 10 s, primer annealing for 15 s, extension at 72 °C for 30 s, and finally, a melting-curve step (95 °C for 15 s, 60 °C for 1 min, and 95 °C for 15 s). Table 1 shows the primers and annealing temperature of the carbapenemase- and virulence-related genes. The mRNA expression levels of the target genes were normalized against those of *16S rRNA*, a housekeeping gene, and the 2^−^^∆∆^^CT^ formula was used.

### 4.8. Statistical Analysis

All statistical analyses in this study were performed with GraphPad Prism 5.0 software (La Jolla, San Diego, CA, USA). The data are presented as means ± standard deviations (SDs). qPCR data were analyzed by the Student’s *t*-test. Statistical significance was considered at * *p* < 0.05, ** *p* < 0.01, and *** *p* < 0.001.

## Figures and Tables

**Table 1:** Minimum inhibitory concentration of fingolimod and carbapenems against *E. coli* strains.

Strains	MIC (μg/mL)				
	Fingolimod	Doripenem	Meropenem	Imipenem	Ertapenem
CLSI breakpoints *E. coli* KBN12P05816	16	≥4 64	≥4 16	≥4 ≥4	≥2 64
*E. coli* KBN12P05795 *E. coli* KBN12P06081	16 16	128 128	16 16	≥4 ≥4	32 32

MIC: minimum inhibitory concentration, KBN: Korea Biobank Network.

**Table 2:** Results of the fingolimod and doripenem combination on CREC strains.

Strain	MIC (μg/mL)			Synergistic Action	
	Fingolimod	Doripenem	Fingolimod + Doripenem	FICI	Interpretation *
*E. coli* KBN12P05816	8	64	4 + 4	0.3125	S
*E. coli* KBN12P05795	16	128	4 + 32	0.5	S
*E. coli* KBN12P06081	16	128	4 + 32	0.5	S

FIC: fractiFIFIC: fractional inhibitory concentration, * S: synergism, I: indifference.

**Table 3:** PCR primers used in this study.

Primers	Target Gene	Primer Sequence (5′-3′)	Annealing Temp. (°C)	Reference
PCR primers	*bla*_KPC_	F: CGTCTAGTTCTGCTGTCTTG	54	[24]
		R: CTTGTCATCCTTGTTAGGCG		
qPCR primers	*acrB*	F: CGTACACAGAAAGTGCTCAA	51	[25]
		R: CGCTTCAACTTTGTTTTCTT		
	*acrD*	F: GCCGTGCAGCAAGTACAAAA	58	[26]
		R: GTATCGCCGGTTTTACGCAC		
	*flhD*	F: ACTTGCACAGCGTCTGATTG	55	[27]
		R: AGCTTAACCATTTGCGGAAG		
	*motA*	F: ACAGGTAGCGCGTTCTCACT	58	[28]
		R: AGCGTGGATAAACCGATACG		
	*bla_KPC_*	F: GATACCACGTTCCGTCTGG	57	[29]
		R: GCAGGTTCCGGTTTTGTCTC		

## Data Availability

All data are presented in the manuscript.
